# Beyond crisis and grief: Rethinking conservation narratives

**DOI:** 10.1093/biosci/biaf017

**Published:** 2025-02-19

**Authors:** Marco Malavasi

**Affiliations:** Department of Chemistry, Physics, Mathematics, and Natural Sciences, University of Sassari, Sassari, Italy

**Keywords:** transformative change, crisis, ecological grief, resignation loop

## Abstract

Since 1985, conservation science has been unquestioningly described as a crisis discipline. This narrative entails prioritizing immediate responses to threats despite limited knowledge. Although crisis interventions have grown significantly, biodiversity loss has continued unabated, and those working or living alongside declining ecosystems report increasing levels of emotional distress. Ecological grief is particularly on the rise and is claimed by some as a necessary process to fuel the urgent lifesaving changes. However, I argue that both narratives—the emerging ecological grief and the established crisis approach—can synergically reinforce a loop of resignation, where ecological decline is either passively accepted or inadvertently perpetuated. Such resignation ultimately leads to harmful adaptation to ongoing ecological degradation. Finally, I propose a shift toward a transformative conservation narrative, moving away from the primary focus on crisis thinking to embrace proactive futures. Reframing the underlying narratives is essential, because they can influence the broader conservation agenda.

In his foundational article, Michael Soulé (1985) asked “What is conservation biology?” paving the way for a new synthetic discipline that aims at addressing the dynamics and problems of perturbated species, communities, and ecosystems. He explains how conservation biology differs from most other biological sciences in one way: It is a crisis discipline; he compares conservation biologists with medical doctors, who are often called on to act rapidly before knowing all the facts.

Since then, conservation biology has considerably evolved (Mace 2014, Evans 2021) and has adapted to the emerging challenges of our time. Kareiva and Marvier (2012) revisited the core principles of conservation biology in favor of a more systemic and evidence-based approach of *conservation sciences*, with a wider range of disciplines to be included. This expanded view acknowledges that conservation actions contribute to more than just biodiversity protection; they can also advance environmental justice (Martin et al. 2016), improve environmental quality, and support transformations in socioeconomic systems, and human health (IPBES 2024). Generally, researchers are becoming self-critical, responsive, and adaptable (Montana et al. 2020); for instance, recognizing that ecological assumptions have been so far shaped and held back by exclusionary Western society, often excluding diverse peoples inhabiting Earth's varied ecosystems (Malavasi 2020, Nuñez et al. 2021, Trisos et al. 2021).

Despite ongoing changes, the narrative of conservation sciences as a crisis discipline remains, to date, unquestioned and widely accepted (Meine et al. 2006, Kareiva and Marvier 2012, Mace 2014, Evans 2021, Stirling and Burgman 2021). The prevailing view holds that urgent and decisive actions before knowing all the facts are essential to reverse global biodiversity loss.

Since 1985, not only have the framing and goals of conservation evolved, but the state of nature has also dramatically shifted. Despite tremendous growth in the volume of crisis interventions and some local successes, the decline of life continues unabated and shows no clear signs of improvement (Williams et al. 2020). Ecological losses are ongoing and cumulative and are uncertain in terms of when, where, and how they will occur (Cooke et al. 2024). We are now grappling with accelerating biodiversity loss, and the window to reverse global warming is rapidly closing. The magnitude of ecological challenges can be overwhelming even for the most optimistic.

In the context of environmental degradation, the levels of emotional distress (e.g., anxiety, worry, or grief) are markedly increasing among children and young people (Hickman 2024) and within the conservation community, especially among people who live and work alongside declining ecosystems (Ojala et al. 2021). Scientists feel increasingly compelled to express their emotions, challenging the traditional expectation of objectivity and neutrality and viewing this openness as crucial to advancing real ecological action (Schipper et al. 2024). Recently, grief over degrading ecosystems is particularly on the rise, leading to the emergence of the term *ecological grief* (Cunsolo and Ellis 2018, Cunsolo et al. 2020, Pihkala 2020, Comtesse et al. 2021, Gerber et al. 2023, Cooke et al. 2024)—the grief felt in relation to experienced or anticipated ecological losses, including the loss of species, ecosystems, and meaningful landscapes because of acute or chronic environmental change. Many scholars are suggesting that mourning and grieving for ecological losses are necessary (Butler 2004, Cunsolo et al. 2020, Varutti 2024). Although grief is uncomfortable, it is claimed that ecological grief is, in fact, the crucible through which humanity must pass to harness the energy and conviction that are needed for the lifesaving changes now required (Butler 2004, Cunsolo et al. 2020, Cooke et al. 2024, Varutti 2024).

Within this context, I argue that, after 40 years, the narrative of conservation sciences as predominantly a crisis discipline requires critical reassessment. Although crisis interventions actively respond to impending threats, their relentless application carries several downsides, including the risk of fostering a loop of resignation that ultimately normalizes adaptation to degrading ecological conditions. Similarly, although emotional distress stemming from environmental degradation must be acknowledged and addressed, I argue that framing ecological grief as a transformative force risks deepening resignation, reinforcing passive acceptance of ecological decline. By highlighting the dangers of entering a loop of resignation fostered by the crisis narrative and the acceptance of grief, I advocate for a shift toward a transformative narrative—conservation sciences as transformative discipline—where prioritizing an epistemological shift allows us to break free from crisis-oriented thinking and envision a radically different future.

Given that human brains quite literally process the world through narratives (Lakoff 2010, Dahlstrom 2014), questioning our underlying narratives is crucial (Louder and Wyborn 2020). Narratives shape how problems are defined influencing the flow of laws, policies, and funding streams (Shanahan et al. 2011). The drive for such a narrative shift could arise from both negative emotions (such as anxiety, fear, and worry) associated with ecological decline, which can be harnessed and transformed into powerful catalysts for action, and a vision of new relationships between people and nature.

## The downside of the crisis discipline narrative

The crisis discipline narrative in conservation science was established to address urgent environmental challenges, emphasizing immediate action. However, keeping this approach has significant downsides. In this section, I first discuss its historical roots in survival science and its reliance on reactive medical analogies. I then examine how its focus on urgency can overshadow systemic thinking, reinforce competition, and favor short-term fixes over transformative change. Finally, I address the emotional toll of perpetual crisis framing, which can lead to resignation and reduced capacity for long-term solutions.

The crisis discipline narrative emerged within the broader context of survival science, which developed from a countercultural movement in the 1970s (Egan 2017). The necessity for survival science became apparent when traditional scientific knowledge, as was noted by Alvin Weinberg (1972), was found inadequate in addressing critical social needs, such as the emerging environmental degradation. This inadequacy is especially apparent in conservation, where evaluating decisions is complicated by the need to account for diverse peoples, cultures, and biodiversity (Pascual et al. 2021). In such complex contexts, effective decision-making requires thoughtful deliberation that goes beyond mere calculations (Stirling and Burgman 2021). These difficulties align with the principles of postnormal science, which emphasize decision-making under conditions of uncertainty, value conflict, and high stakes (Funtowicz and Ravetz 1990).

In response to these challenges, Soulé (1985) introduced *crisis discipline* as a framework for conservation science, deliberately drawing on medical analogies to highlight the urgency of intervention—often taken without complete knowledge—with the word *crisis* itself rooted in historical and medical contexts. The word *Kρίσις* (*krìsis*), deriving from the Greek verb *κρίνειν* (*krìnein*), originally signified the process of separating wheat from chaff and straw, leading to a decisive judgment (Chantraine [1968] 2009). This term was soon adopted by the Hippocratic school (the Corpus Hippocratum) in the fifth century BCE to describe a critical medical phase—a decisive point where life or death hung in balance, demanding swift, irrevocable intervention (Roitman 2014). It is in this very idea of finite, urgent, and high stakes intervention that conservation science finds its foundation. In parallel to medical practice, conservation actions are meant to be immediate, reactive efforts aimed at mitigating damage. Soulé (1985) himself clearly underscores this analogy, when he states that conservation biology is related to ecology in the same way that surgery is related to physiology or war is to political sciences. He also draws further parallels with cancer biology, describing both as synthetic, multidisciplinary fields that tackle complex issues. In any case, urgent decisions must be taken before knowing all the facts; crisis disciplines are therefore a mixture of science and art (Soulé 1985)*.* This often implies neglecting broader systemic implications in the pursuit of minimizing losses before they become irreversible.

However, 50 years after the crisis narrative’s inception, it is crucial to assess how the analogies used in Soulé (1985) proved to be relevant and effective when applied to the unrelenting ecological tragedy. Notably, the ecological crises can no longer be intended as a finite moment of decisive and urgent judgment (e.g., performing a surgery or entering a war) where swift reaction can solve the question at hand. Instead, the ecological crisis is now posited as a protracted and potentially persistent state of ailment and demise (Roitman 2014). Koselleck (1988) emphasized that addressing such protracted crises requires not only immediate action but, more importantly, a transformative change in our epistemological approach. This means reevaluating and restructuring how we understand and respond to these persistent challenges to create more effective solutions. On the contrary, characterizing conservation sciences primarily as crisis intervention oriented may hinder the necessary flexibility and foresight needed to address the required change (Evans et al. 2017, Montana et al. 2020). The need for such an epistemological change is in line with what was recently highlighted in the Intergovernmental Science-Policy Platform on Biodiversity and Ecosystem Services’ (IPBES) Global Assessment (IPBES 2019), which called for an urgent and proactive transformative change, defined as fundamental system-wide shifts in views (ways of thinking, knowing, and seeing) structures (ways of organizing, regulating, and governing), and practices (ways of doing, behaving, and relating). Transformative change must address the root causes of biodiversity loss—interconnected indirect drivers such as economic, sociocultural, demographic, political, institutional, and technological forces that underpin direct drivers such as land and sea use change, the exploitation of organisms, climate change, pollution, and invasive species (Díaz et al. 2019, Fischer and Riechers 2019, Wyborn et al. 2020). These indirect drivers are shaped by dominant values and epistemologies that manifest through patterns of consumption, trade, governance, conflicts, and technological advancements.

Conversely, the inherent risk in the crisis narrative is its emphasis on immediate interventions, which often leads to a loop of symptom management. Although this reactive approach is reasonable under specific circumstances, it may also lead to overlooking indirect drivers or patterns and cycles in systems, crowding out opportunities to reflect on the broader systemic implications of our actions (Capra and Luisi 2014). The urgency of action discourages deep thinking—often termed *slow science*—and prevents critical reevaluation of the fundamental goals and roles that guide conservation science (Stengers and Muecke 2017, Turnhout and Lahsen 2022). Conversely, this rush to act prioritizes speed and productivity, sidelining the slower, more deliberate approaches required to deeply understand and address ecological complexity. Worse still, the crisis narrative can obscure rising individualism and competition within the scientific community (Büscher et al. 2012), because these behaviors are hidden behind a facade of commitment to ecological stewardship. This mindset risks creating the illusion that securing project funding or publishing papers is equivalent to genuinely addressing ecological challenges, reinforcing performative rather than transformative actions. Similarly, the focus on urgent action may reinforce the complacent positivist beliefs often held by environmental scientists that scientific results are neutral and value free (Barry and Oelschlaeger 1996, Roebuck and Phifer 1999) and that a strict implementation of such results to other areas of society certainly leads to the resolution of the environmental tragedy (the loading-dock approach; Rogga 2020). Unfortunately, too often, conservation scientists remain reluctant to recognize the normativity of the field. This model of science–policy relationship constrains the avenues for receiving feedback and encouraging critical inquiries, further entrenching the cycle of crisis management.

Most importantly, although crisis interventions are essential in certain moments, relying on them predominantly can lead to a resignation loop, which ultimately means adapting to the altered circumstances of highly toxic ecosystems (see the “The harmful loop of ecological resignation” section). The relentless focus on immediate short-term responses falls short when addressing the scale and complexity of contemporary ecological issues. Stakeholders may come to accept environmental degradation as inevitable after repeatedly attempting urgent actions that ultimately fail to resolve the underlying issues. This resignation can grow increasingly comfortable over time, resembling a form of emotional self-preservation. Remaining within the familiar cycle of crisis management, reinforced by the comforting self-reassurance that we are taking action, feels safer than risking failure in the pursuit of a challenging transformative change (Stokes 1962). Just as one might choose not to stand up after being knocked down to avoid further blows, sticking to the crisis narrative can shield us from the disillusionment that often follows repeated failures but at the cost of perpetuating a sense of numbness and helplessness. These emotions are well documented within the scientific community: psychic numbing allows researchers to distance themselves from ongoing loss (Norgaard 2011) while they increasingly leave the field in numbers, citing feelings of futility, grief, and despair (Clayton 2018, Pihkala 2020) or profound hopelessness (Fraser et al. 2013, Head and Harada 2017).

In *Anti-Crisis*, Janet Roitman (2014) effectively encapsulated the risk of crisis interventions, particularly the tendency to foster resignation and adaptation to the actual circumstances. Roitman (2014) argued that such urgent short-term interventions limit the scope of possible actions and stifle deeper exploration of systemic issues. In the loop of crisis interventions, the same structures that contribute to the problems remain unchallenged and reinforced. By continuously responding to emergencies, crisis interventions can trap stakeholders in a cycle that undermines the potential for meaningful progress, leaving little room for hope or transformative change (Masco 2016).

## The downside of ecological grief

In August 2019, Iceland held a symbolic funeral for Okjökull, the first of its glaciers lost to climate change. This unprecedented event, attended by scientists, activists, and politicians, gained global attention for drawing public focus to the tangible effects of global warming (Craps 2023). A plaque titled *A Letter to the Future* was unveiled, stating, “Ok is the first Icelandic glacier to lose its status as a glacier. In the next 200 years, all our glaciers are expected to follow the same path. This monument is to acknowledge that we know what is happening and what needs to be done. Only you know if we did it.” The funeral not only commemorated a vanished glacier but also blended ecological grief with a call for collective responsibility. It has since inspired similar ceremonies around the world, highlighting the cultural and emotional dimensions of climate change.

Although ecological grief is still considered a type of disenfranchised grief (Cunsolo and Ellis 2018, Cooke et al. 2024)—in which significant loss is not culturally recognized or supported—the topic of ecological grief has emerged in mainstream academic literature only in the last few years so that a new topic of ecological grief literacy is born (Cooke et al. 2024). This is defined as the knowledge, skills, and values that promote the understanding of and action toward providing compassionate support for those experiencing ecological grief, including ourselves.

Varutti (2024) recently emphasized the moral imperative of ecological grief and mourning. It has also recently been claimed that grief is the only way to deal productively with the negative emotions—ecoanxiety, worry, a lack of hope—that paralyze us. Acknowledging our ecological grief and staying with the loss can bring to light new meanings, enhance personal resilience, and sustain effective environmental action (Cunsolo et al. 2020, Pihkala 2020, Cooke et al. 2024). As was discussed by Butler (2004), grief and mourning highlight our deep connections to ecological communities and reveal our ethical responsibilities toward them. According to thinkers such as Butler (2004), these emotions have *we*-creating capacities, uncovering hidden connections between individuals and offering opportunities to bridge differences. In essence, grief and mourning challenge our assumptions about what we value and consider worthy of grief, reshaping our understanding of knowledge and loss.

Nonetheless, it must be noted that research on ecological grief is still emerging (Cunsolo and Ellis 2018, Comtesse et al. 2021, Cooke et al. 2024), and little is known about the political and social implications of coming to terms with ecological grief. To grasp these aspects, it may be helpful to delve into the biopsychological significance of the grief process. So far, among the most acknowledged key theories of grief, we have Kübler-Ross and colleagues’ (1972) five stages of grief and Stroebe and Schut's (1999, 2010) dual process model. These theories were originally developed for coping with the death of a partner, but they can also apply to other forms of loss and trauma.

Kübler-Ross and colleagues (1972) outlined five stages of grief: denial, anger, bargaining, depression, and acceptance. These stages are not necessarily experienced in a linear progression, and not everyone will encounter all five stages. Although each stage serves a distinct and essential role, the ultimate outcome is integrating the loss into one's life narrative, resigning to the irreversible loss to adapt to the altered circumstances of our lives. In contrast, Stroebe and Schut's (1999, 2010) dual process model presents a more dynamic approach. It posits that individuals oscillate between two types of coping: loss-oriented coping, which is focused on grieving the loss, and restoration-oriented coping, which addresses the changes and new demands following the loss. Acceptance, in this model, involves striking a balance between these two processes, because individuals find new meaning while accepting the irreversible loss and the new circumstances(Bennett et al. 2010).

Regardless of the framework used, grief models agree on the idea that grief involves an acceptance of both experienced and anticipated losses, acknowledging the irreversibility of what has been lost. Although this acceptance can help alleviate the psychological distress and burnout related to ecological loss (Cunsolo and Ellis 2018, Pihkala 2020, Verlie et al. 2021)—an essential function of grief—it automatically leads to adapting to and normalizing new—often harmful—environmental conditions. Consequently, engaging in the grief process may diminish the drive to continue fighting against significant ecological losses. It can also lessen the fear and distress that often fuel conservation efforts, potentially dulling the sense of urgency required for meaningful political and social engagement.

Moreover, the rise of this type of distress suggests that many individuals facing this ecological grief are already turning to biomedical healthcare systems for help and treatment (Wardell 2020). The medicalization of grief can be an even more dangerous step, because it shifts the narrative from addressing systemic environmental failures to framing individuals as ill, convincing us that we need healing instead of action (Wardell 2020). Wardell (2020) reminded us that such categories of distress are not universal but are culturally constructed, shaped by prevailing power dynamics that seek to control emotions and preserve the status quo. Contextualizing ecological grief as a phenomenon of our time reminds us that it is neither absolute nor inevitable and that it can be challenged to avoid reinforcing resignation.

Finally, rather than viewing ecological grief as something to be healed and experienced, we must consider the option to resist such resignation and medicalization in order to keep distress (e.g., anxiety, fear, worry) alive as a driving force for sociopolitical engagement and action. If we accept grief and resign ourselves to ecosystem degradation and if the prospect of meaningful action is abandoned, we may as well prepare for palliative care—passively awaiting the inevitable while numbing ourselves with painkillers.

## The harmful loop of ecological resignation

**Figure 1.**
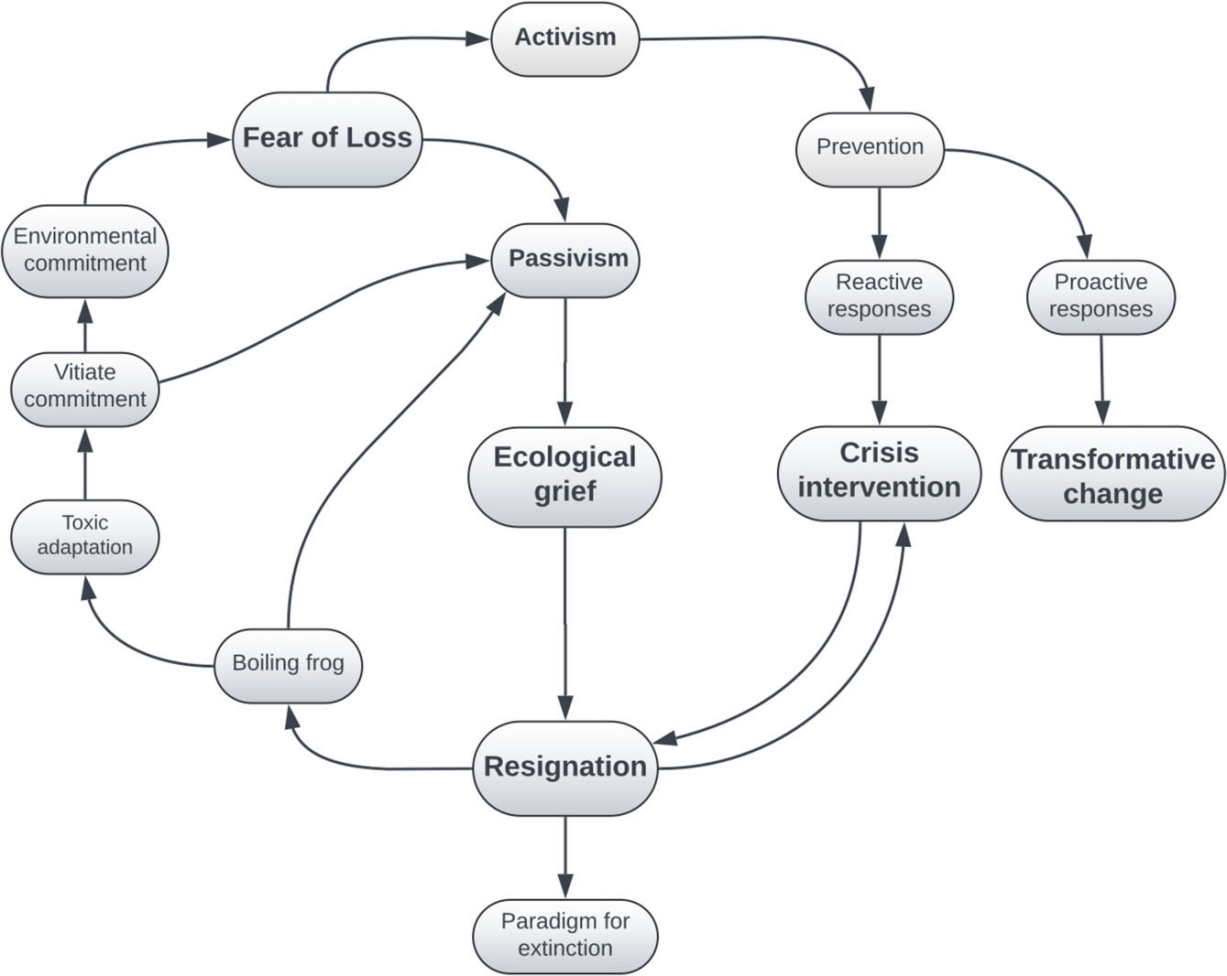
The loop of ecological resignation illustrates how the crisis narrative traps us in cycles of fear-driven reactions. Fear can lead either to energized activism or to passive grief, where acceptance of ecological loss breeds maladaptive coping, disconnection, and toxic adaptation. Even active responses risk fueling this loop, numbing commitment, and perpetuating ecological decline.

The loop of ecological resignation presented in figure 1 illustrates the risks of relying predominantly on reactive measures, as is emphasized by the crisis narrative, and entering an ecological grief process. This framework serves as a warning against becoming trapped in a cycle of resignation, where ecological decline is either passively accepted or inadvertently reinforced, even through seemingly active responses.

The experience and acknowledgment of the risk posed by the current losses or anticipated future changes can generate negative emotions, such as worry, fear, and anxiety. Worry is described as a complex emotion that evolves from more basic feelings such as fear and anxiety (Power and Dalgleish 2015). Fear is an immediate emotional response to a perceived threat, triggering instinctive fight or flight reactions. In contrast, anxiety combines emotional and cognitive elements, making it a future-oriented response linked to uncertainty. Worry further builds on anxiety, emphasizing cognitive processes that anticipate risks and stimulate problem-solving efforts (Sweeny and Dooley 2017). Despite these differences, fear, anxiety, and worry are all normal, adaptive reactions to environmental threats, especially when individuals have adequate resources to manage them (Ojala et al. 2021).

Indeed, such emotional distresses are known to represent a sign of relationship with and connection to the natural world (Cunsolo et al. 2020, Ojala et al. 2021). Among these, I focus on fear, using it as a fundamental and basic feeling that forms the foundation of more complex emotional responses. When confronted with fear, our reaction can bifurcate into two radically different streams: passivism and activism.

The passive stream leads into the processes of grief, which serve as a biopsychological means of coping with unavoidable loss, either experienced or anticipated. Entering the grief domain is often an automatic response to accepting the inevitability and irreversibility of how our experiences have been radically changed (Kübler-Ross et al. 1972, Stroebe and Schut 2010). This acceptance removes the possibility or necessity of continuing to struggle against such profound change, resulting in a sense of defeat and resignation to our impotence in restoring or reinstating our previous state. This resignation means we must adapt to the altered circumstances of our lives (i.e., ecological loss).

Because the changes in the ecosystems are highly toxic, this adaptation involves navigating toxic interactions that have damaging effects on our well-being (i.e., maladaptive response). These harmful effects put us in the model of the boiling frog—a metaphor suggesting that if a frog is placed in cool water that is gradually heated, it will fail to perceive the danger and remain in the pot, ultimately boiling to death, whereas if placed suddenly in hot water, it would instinctively jump out. Although not confirmed by scientific experiments, this metaphor illustrates how organisms, including humans, may fail to recognize or respond to gradually worsening conditions until it is too late. Several maladaptive responses to ecological losses have been reported within the scientific community (Pihkala 2020). In addition to psychic numbing and pervasive feelings of hopelessness and helplessness, Hoggett and Randall (2018) showed that this maladaptive coping mechanism can be socially reinforced. People at the workplace may create a jointly upheld atmosphere and belief that there are no problems at hand. Similarly, researchers are trying to be constantly busy, workaholism being one of the most common responses to ecological loss (Cunsolo Willox et al. 2013). The crisis narrative perfectly fuels this coping mechanism, because its sense of urgency drives relentless activity, numbing the despair associated with environmental degradation. Burnout among researchers and practitioners is also on the rise (Fraser et al. 2013, Gerber et al. 2023). The overall effect of this stream is to vitiate commitment, weakening it and fostering a sense of disconnection from one's ecosystem. If this loop becomes overpracticed, it can ultimately lead to the paradigm of extinction. As was observed by Bateson (1972), the species that destroys its medium because of a disconnection from the ecosystem is on its way to extinction.

The activist stream enhances commitment, raises the level of risk we are willing to assume, increases awareness of the threat of loss, and fuels political and scientific activism aimed at conserving what we are not prepared to lose. This drive fosters both proactive and reactive measures. The reactive responses involve crisis interventions that prioritize managing crises with the risk of not addressing their root causes. As such, this reactive approach can easily lead to resignation and the boiling frog phenomenon of toxic adaptation, resulting in maladaptive responses, demoralization, and disconnection. Proactive responses demand transformative changes, which require an epistemological shift: redefining human–nature relationships and reshaping our approach to address ecosystem degradation while promoting sustainability (Díaz et al. 2019). Among the proactive strategic initiatives, also the nature positive framework is emerging, which aims for measurable improvements in ecosystem health, abundance, diversity, and resilience from a 2020 baseline (Maron et al. 2024). Although conservation agendas have traditionally been focused on merely mitigating impacts or avoiding, minimizing, and compensating for harm to biodiversity, the idea of nature positivity extends this approach by requiring measurable, absolute improvements in biodiversity over time (Maron et al. 2024). Crucially, implementing nature positivity necessitates robust net gain legislation to facilitate a tangible reversal of biodiversity decline (Thomas et al. 2024).

## Ways forward

The crisis discipline narrative undeniably played—and continues to play—a crucial role in shaping our understanding of environmental issues and driving urgent policies and actions (Louder and Wyborn 2020). Nonetheless, I argue that framing conservation primarily as a crisis discipline can create a loop of resignation and symptom management, despite its roots in a proactive engagement with the environment.

Similarly, although ecological grief as a legitimate response to ecological loss deserves formal recognition (Comtesse et al. 2021), I contend that promoting grief—particularly as an anticipatory process—as the sole or primary means of constructively processing negative emotions such as fear, worry, or anxiety can inadvertently reinforce a cycle of resignation.

This resignation forces us to adapt to harmful ecological changes, akin to the boiling frog effect, where ongoing adaptation to worsening conditions harms our well-being. Over time, this leads to reduced or vitiate commitment, demotivation, and disconnection from the ecosystem.

On the contrary, negative emotions such as fear, worry and anxiety have historically been a powerful catalyst for human action (Weber 2006), and they can similarly serve as powerful motivators for proenvironmental behavior. Such emotions can help ensure that these harmful ecological changes are not normalized; that is, the idea of the new normal should not include accepting the loss of species, habitats, ecosystem function, or human lives (Tschakert et al. 2017, Schipper et al. 2024). However, to harness their potential effectively, we need accessible and supportive spaces for processing and sharing these emotions, strong political commitment to fund essential strategies, and research dedicated to developing resilience-enhancing approaches (Cunsolo et al. 2020). It is also important to differentiate between ecological grief and concepts of emotional distress such as fear, anxiety, and worry (Comtesse et al. 2021).

Finally, I propose a more comprehensive narrative in the conservation sciences that acknowledges the importance of reactive strategies (i.e., crisis interventions) but emphasizes proactive measures (e.g., transformative change or nature positive goals) as the dominant approach. Given the urgency of the current situation, it is essential to emphasize transformative change as the guiding narrative—reframing conservation science as a transformative discipline.

Practically speaking, no silver bullet can reverse the self-destructive trends that have culminated in the proclamation of the Anthropocene (Fischer and Riechers 2019). Nonetheless, drawing on systems theory (Meadows 1999), we can identify leverage points as strategic positions within complex systems where small shifts can trigger significant, cascading transformative changes. These leverage points range from adjusting system parameters to transforming paradigms, with interventions becoming more effective as they target deeper changes in narratives and worldviews. Building on this foundation, the transformative change proposed by the IPBES (2019) aligns closely with systems thinking, identifying several leverage points at multiple levels (see Díaz et al. 2019)—from modifying incentives and regulations to reshaping values and worldviews.

Changing the conservation narrative from a crisis to a transformative narrative represents a critical leverage point within this framework. Such a new narrative should consider conservation action to contrast the disconnection of people from nature, domination over nature and other people, inequitable concentrations of power and wealth, and the prioritization of short-term individual and material gains. These interventions should be guided by four principles: equity and justice, pluralism and inclusion, respectful and reciprocal human–nature relationships, and adaptive learning and action.

The IPBES (O'Brien et al. 2024) also highlights the importance of visions of a sustainable world for nature and people to inspire transformative change. Imagination and creativity are emphasized as essential tools to foster new ways of addressing longstanding problems and creating better futures (Wyborn et al. 2020).

In this context, the concept of the Ecocene recently proposed by Tanasescu (2022) can represent a new vision to support such transformative change. It emerges as a hopeful and forward-looking alternative to the Anthropocene, shifting away from narratives centered on human dominance and ecological crisis. Although the Anthropocene highlights humanity's profound and often destructive impact on the planet, the Ecocene envisions a future of coexistence and ecological awareness. It promotes breaking free from the reactive, crisis-driven cycle that fosters resignation and helplessness. Instead, the Ecocene encourages proactive engagement, aligning human societies with the adaptive rhythms of nature. By transforming fear and worry into enduring motivation for ecological stewardship, it invites us to cocreate a thriving, interdependent future in harmony with the living world.

## Author Biography

Marco Malavasi (mmalavasi@uniss.it) is affiliated with the Department of Chemistry, Physics, Mathematics, and Natural Sciences at the University of Sassari, in Sassari, Italy.
